# Food and Housing Insecurity, Stress, and Health Care Use After Medicaid Expanded Services Program

**DOI:** 10.1001/jamanetworkopen.2025.19507

**Published:** 2025-07-08

**Authors:** Anne N. Thorndike, Jessica L. McCurley, Yuchiao Chang, Jessica Cheng, Cheryl R. Clark, Christine Vogeli, Sydney McGovern, Vicki Fung, Douglas E. Levy

**Affiliations:** 1 Division of General Internal Medicine, Massachusetts General Hospital, Boston; 2 Harvard Medical School, Boston, Massachusetts; 3 Department of Psychology, San Diego State University, San Diego, California; 4 Department of Epidemiology, Harvard T.H. Chan School of Public Health, Boston, Massachusetts; 5 Division of General Internal Medicine & Primary Care, Brigham and Women’s Hospital, Boston, Massachusetts; 6 Mongan Institute Health Policy Research Center, Massachusetts General Hospital, Boston

## Abstract

**Importance:**

Massachusetts implemented a Flexible Services program (FSP) under a Medicaid Section 1115 waiver to address food and housing insecurity for accountable care organization (ACO) beneficiaries. Little is known about the social, behavioral, and clinical outcomes associated with Medicaid social needs interventions.

**Objective:**

To compare 1-year changes in food and housing insecurity, diet, stress, and acute health care use between individuals who participated in the Massachusetts FSP and those who did not.

**Design, Setting, and Participants:**

This prospective cohort study enrolled adult Medicaid beneficiaries from 5 community health centers in 1 eastern Massachusetts ACO from December 2019 through December 2020. Participants who were enrolled in FSP were compared with propensity score–weighted FSP-eligible study participants who were not enrolled in FSP (hereafter non–FSP participants). All FSP participants completed surveys and dietary recalls at enrollment and then annually for 3 years. In-depth interviews were conducted with 27 FSP participants. All data analyses were conducted from August 2024 to April 2025.

**Exposure:**

Enrollment in FSP.

**Main Outcomes and Measures:**

Primary outcomes were 1-year changes in food and housing insecurity, stress, dietary quality, and acute health care use. Secondary outcomes were changes in depression and anxiety symptoms. Annual surveys measured food insecurity (10-item US Department of Agriculture Food Security Scale Module; score range: 0-10, with ≥3 indicating food insecurity), housing insecurity (questions regarding current housing, moving ≥2 times in past year, and worrying about losing housing), and stress (the 10-item Perceived Stress Scale [PSS]; score range: 0-40, with ≥14 indicating moderate to severe stress). Diet quality was measured with the Healthy Eating Index-2020 (HEI-2020; score range: 0-100, with higher scores indicating healthier diet quality). Acute health care use included annual emergency department (ED) visits and acute hospitalizations.

**Results:**

There were 153 FSP participant episodes (representing 153 study participants; mean [SD] age, 43.6 [10.8] years; 129 females [84.3%]) and 1495 non–FSP participant episodes (representing 610 unique study participants; mean (SD) age 43.2 (11.2) years; 464 females [76.1%]) in the sample. Before FSP enrollment, 111 (72.5%) had food insecurity, 68 (44.4%) had housing insecurity, and 55 (35.9%) had both. There were no differences between the FSP and non-FSP groups in changes in proportion of individuals with food insecurity (difference in change, 4.96%; 95% CI, −3.13% to 13.05%) or housing insecurity (difference in change, 2.75%; 95% CI: −5.39% to 10.88%). There were no differences between groups in HEI-2020 scores, PSS scores, or acute health care use. Participant interviews reflected a range of experiences associated with FSP, from favorable changes in social needs and health to persistent challenges related to cost of food and limited housing availability.

**Conclusions and Relevance:**

This study of adult Medicaid beneficiaries found that FSP was not associated with short-term favorable changes in food or housing insecurity, diet, stress, or acute health care use. In interviews, FSP participants highlighted both the benefits and challenges of addressing social needs through such health system interventions.

## Key Points

**Question:**

What changes in social needs, diet, stress, and acute health care use are associated with implementation of the Massachusetts Medicaid Flexible Services program (FSP) addressing food and housing insecurity?

**Findings:**

In a cohort study of 153 FSP participant episodes and 1495 non–FSP participant episodes among adult Medicaid beneficiaries, there were no differences in food or housing insecurity, dietary quality, stress, or health care use between these 2 groups at 1 year. In interviews, participants reported experiencing a range of positive, neutral, and negative changes following FSP.

**Meaning:**

The findings suggest that FSP participation was not associated with positive changes in social needs, diet, stress, or health care use within the first year, although some participants reported benefits.

## Teaser

This cohort study assesses the social, behavioral, and clinical outcomes associated with participation in the Massachusetts Medicaid Flexible Services program.

## Introduction

Health-related social needs, such as food and housing insecurity, are associated with chronic disease, high health care costs, and health disparities.^1,2,3^ With increasing focus on value-based care and health equity, health systems have invested billions of dollars into social needs programs.^4,5^ As of October 2024, 21 states had received Medicaid Section 1115 waivers to conduct demonstration or pilot projects focused on health-related social needs.^6^ Still, little is known about whether programs are associated with improved key social, behavioral, and clinical outcomes.^7^

Prior studies of health care–based social needs programs have had mixed results. Most were uncontrolled, pre-post studies with limited ability to account for secular trends.^8,9,10^ Data from larger randomized and controlled studies, however, suggest limited impact.^11,12,13,14^ For example, in the Centers for Medicare and Medicaid Services Accountable Health Communities Model, several thousand participants were randomized to navigation services and resource referral or to referral only.^11^ Interim analyses demonstrated the intervention group had fewer emergency department (ED) visits but no differences in hospitalizations, total spending per beneficiary, use of community services, or resolution of social needs.^12,13^ In a California Medicaid housing program study, 845 adults receiving a 1-time rental housing deposit had no change in 6-month health care use compared with matched controls.^14^

Few studies have evaluated the implications of health-related social needs interventions for behavioral outcomes, such as diet and stress, which are mediators of chronic disease.^15,16^ Randomized trials of medically tailored meals and pre-post evaluations of produce prescriptions have demonstrated some dietary improvement.^17,18,19,20,21,22,23^ Although small studies have reported that medical-legal partnerships were associated with decreased stress,^24,25^ a randomized trial of a health care community resource referral intervention demonstrated no improvement in mental health–related quality of life.^26^

As part of a Section 1115 waiver, Massachusetts Medicaid (MassHealth) implemented the Flexible Services program (FSP) in 2020 to address food and housing insecurity among accountable care organization (ACO) beneficiaries.^27^ MassHealth provided $149 million statewide across 17 ACOs to deliver FSP; most of the funding went toward payments from ACOs to social service organizations (SSOs). The program was not a benefit or covered service, and not all FSP-eligible beneficiaries could participate. An interim evaluation of FSP in the Mass General Brigham (MGB) ACO demonstrated several implementation barriers, and only 1.6% of adult beneficiaries were enrolled.^27^ A recent study using administrative data from 20 403 MassHealth FSP nutrition services participants compared with 2108 beneficiaries who were referred but not enrolled found that FSP was associated with decreased hospitalizations and ED visits.^28^

This study, LiveWell/ViveBien, prospectively enrolled adult MGB ACO beneficiaries prior to FSP participation and followed up participants for 3 years. The objective was to compare 1-year changes in food and housing insecurity, diet, stress, and acute health care use between participants who were enrolled in FSP and propensity score–weighted study participants who were eligible but not enrolled in FSP (hereafter non–FSP participants). Qualitative interviews with participants enrolled in FSP provided beneficiaries’ perspectives on FSP outcomes.

## Methods

### Study Design

LiveWell/ViveBien was a prospective cohort study of adults in the MGB Medicaid ACO. Study participants were enrolled between December 2019 and December 2020, prior to FSP enrollment, and were followed up for 3 years. Study participants who were enrolled in FSP during the study were compared with propensity score–weighted study participants who were not enrolled. The MGB Institutional Review Board approved all study procedures on August 27, 2019. Study participants provided verbal informed consent, and interviewees provided consent for use of their interview content. We followed the Strengthening the Reporting of Observational Studies in Epidemiology (STROBE) reporting guideline.

### Participants

Study participants were adults receiving care at 5 community health centers in greater Boston that are affiliated with Massachusetts General Hospital and Brigham and Women’s Hospital. Participants were enrolled in FSP in person and by telephone. To be eligible, an individual had to be a member in the MGB ACO, be 21 to 62 years of age, speak English or Spanish, and have had 2 or more health center visits in the prior 2 years.

### FSP

MassHealth ACOs independently contracted with SSOs to provide nutrition and housing–related services to referred patients. To be eligible for FSP enrollment, individuals needed to be ACO members with food or housing insecurity identified by screening or clinical encounter and meet at least 1 health criterion, including complex physical health need (eg, uncontrolled hypertension), complex behavioral health need (eg, uncontrolled depression), high ED use (ie, ≥2 visits in 6 months or 4 visits in 1 year), and high-risk pregnancy. The ACO referred FSP enrollees to SSOs. Initial enrollment duration was typically 6 months; individuals could be re-enrolled in nutrition services by ACO staff if needed. Nutrition SSOs provided supermarket produce vouchers, food boxes, medically tailored meals, and nutrition education. Housing SSOs provided pretenancy and posttenancy support, including assistance with housing applications, searches, and legal support. The MGB ACO began FSP enrollment in March 2020.^27^

### Measures and Outcomes

Study participants completed a survey and 2 Automated Self-Administered 24-hour (ASA24) Dietary Assessment Tool recalls^29^ at enrollment (baseline) and 3 annual follow-ups, either online or by telephone. The survey questions asked about demographics (including race and ethnicity), social needs, mental health, and health behaviors. Race and ethnicity were self-identified on study surveys and included the categories Hispanic, non-Hispanic Black, non-Hispanic White, and other (including American Indian or Alaska Native, Asian, Native Hawaiian or Pacific Islander, multiracial, and other race [not defined]). Race and ethnicity data were included in analyses to account for cultural preferences and norms that may have affected study outcomes, including experience of social needs and stress, dietary intake, and acute health care use. Diagnoses and health care use were collected from electronic health record (EHR) and claims data. Enrollment in FSP was determined using data from the EHR, health system and SSO data-sharing platform, and ACO quarterly reports.

Primary outcomes were 1-year changes in food and housing insecurity, stress, dietary quality, and acute health care use. Secondary outcomes were changes in depression and anxiety symptoms. Food insecurity was assessed with the 10-item US Department of Agriculture Food Security Scale Module (score range: 0-10), which was dichotomized so that a score of 3 or higher (low or very low food security) indicated food insecurity. Housing insecurity was ascertained from agreement with at least 1 of 3 questions: currently does not have own housing, moved 2 or more times in the past year, and worried about losing housing in the next 2 months. Stress was assessed with the 10-item Perceived Stress Scale (PSS; score range: 0-40, with ≥14 indicating moderate to severe stress).^30^ Depression and anxiety symptoms were assessed using the 8-item Patient Health Questionnaire Depression Scale (PHQ-8; score range: 0-24, with ≥10 indicating moderate to severe depressive symptoms) and the 7-item Generalized Anxiety Disorder (GAD-7; score range: 0-21, with ≥10 indicating moderate to severe anxiety symptoms).^31,32^ Dietary quality measured with the Healthy Eating Index-2020 (HEI-2020; score range: 0-100, with higher scores indicating healthier diet quality)^33^ was calculated for each time point using ASA24 dietary recalls. Higher HEI-2020 total score indicates a healthier diet, and the total score is made up of 9 adequacy (eg, total vegetables) and 4 moderation component (eg, sodium) scores for food groups and nutrients. The mean number of acute health care visits in the prior 12 months, including ED visits and unplanned hospitalizations (excluding normal pregnancy, planned surgeries, and rehabilitation stays), was assessed at each time point.

### Analytic Sample

The analytic sample included LiveWell/ViveBien study participants enrolled in FSP and FSP-eligible ACO members with eligible diagnosis not enrolled in FSP (Figure). To be included in the sample, FSP participants had to have completed a survey in the year prior to their enrollment (time 0), completed a survey at the subsequent 12-month follow-up (time 1), and been enrolled in FSP between time 0 and time 1. For example, a participant who was enrolled in FSP after completing their baseline survey but before completing their 1-year follow-up survey would be included in the sample if they had completed both the baseline and 1-year surveys; if this participant did not complete a 1-year survey, they would be excluded. Only the first FSP enrollment per participant was included.

**Figure.**
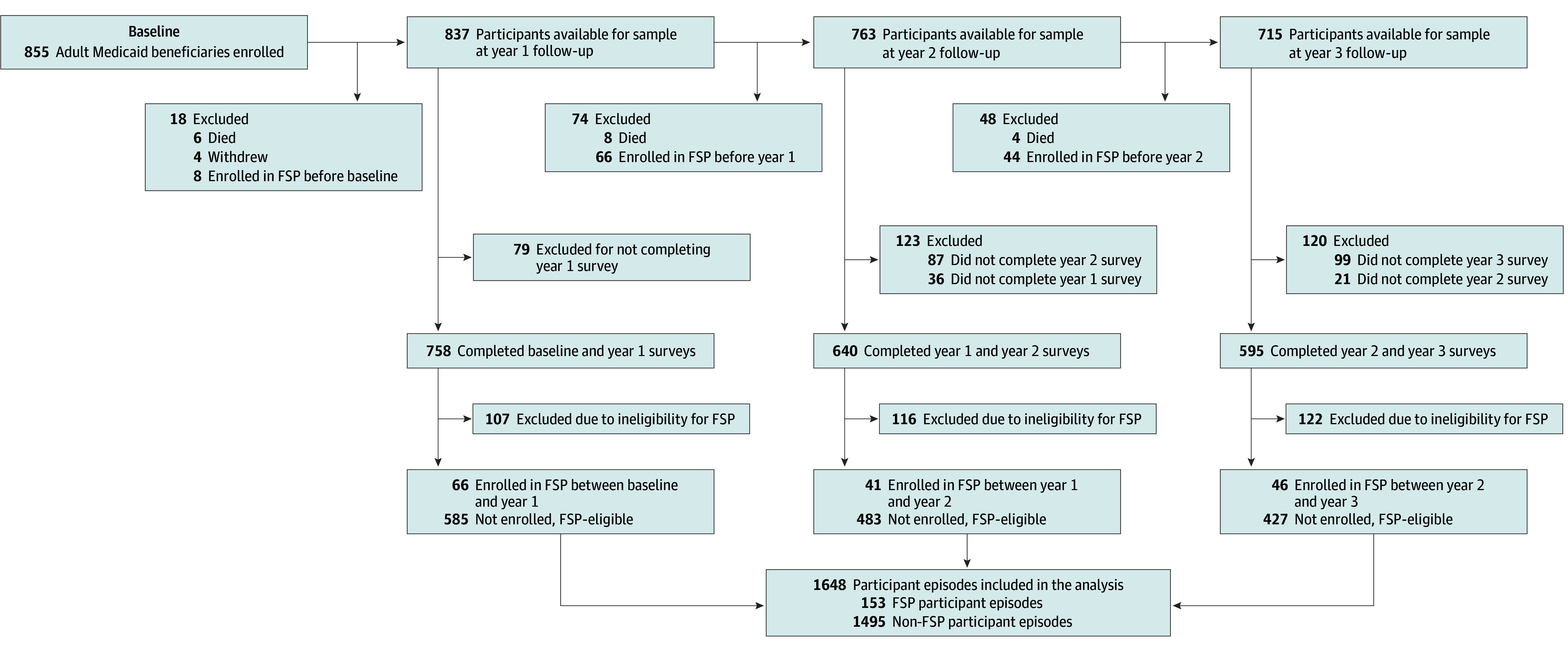
Study Flow Diagram of Participants Included in the Flexible Services Program (FSP) and Non-FSP Groups in the Analytic Sample FSP-eligible indicates a participant in the Medicaid accountable care organization, with food or housing insecurity, and with a diagnosis that would fulfill criteria for program enrollment.

To be included in the sample as a non–FSP participant, an individual had to have never been enrolled in FSP, have been FSP-eligible in the 12 months prior to time 1 (confirmed Medicaid ACO insurance, prior 12-month food or housing insecurity based on time 1 survey questions, and FSP-eligible diagnosis documented in the EHR during the prior 12 months), and have completed surveys at both time 0 and time 1. Non–FSP participants included at one time 1 could be included in future follow-up time points, either as a new FSP participant or again as a non–FSP participant.

### Qualitative Interviews and Thematic Analysis

Study participants who provided qualitative interviews were current or prior FSP participants, purposively sampled so that their range of characteristics (sex, race and ethnicity, primary language, health center affiliation, and service type [nutrition, housing]) reflected the overall study cohort. Interviews were conducted in English or Spanish using a semistructured interview guide that included questions about perceived effectiveness of FSP in addressing food or housing needs, diet, stress, and health. Interviews were conducted by telephone, audiorecorded, transcribed, coded deductively using Dedoose software, version 9.0 (SocioCultural Research Consultants LLC), and analyzed (using the Framework Method for structured thematic analysis).^34^ Three trained research assistants (2 of whom were bilingual) coded all transcripts, and a PhD-level researcher (J.L.M.) resolved discrepancies. Saturation was determined when novel themes no longer emerged. Themes and results were reviewed with the research team to ensure face validity.

### Statistical Analysis

Propensity score weighting was used to balance the non-FSP group against the FSP group based on calendar year and sociodemographic and clinical characteristics at time 0, collected from surveys, dietary recalls, EHR, and claims.^35^ Standardized mean differences were used to measure the balance of individual covariates after weighting. The FSP and weighted non-FSP groups were compared on 1-year changes (time 0 to time 1) by testing the time-by-group interactions in logistic regression models for the proportion of participants with food insecurity and the proportion of participants with housing insecurity; in linear regression models for HEI-2020, PSS, PHQ-8, and GAD-7 scores; and in Poisson models for ED visits and hospitalizations. Generalized estimating equations were used to account for repeated measures from the same participants at multiple time points.

Three sensitivity analyses were conducted. The sample for the first sensitivity analysis was limited to FSP participants receiving nutrition services, enabling an assessment of whether changes in outcomes were greater after excluding those who received only housing services. The second sensitivity analysis sample was limited to current FSP participants in nutrition services at time 1, allowing an assessment of whether changes in outcomes were greater when individuals were actively receiving services in contrast to the first sensitivity analysis that included both participants actively receiving and recently completing services. The third sensitivity analysis sample was limited to FSP participants who had participated in the program for 6 months or longer at time 1. Radar plots were used for visual analysis of HEI-2020 component scores at time 0 and time 1 for FSP participants in nutrition services and non–FSP participants.

The original power calculation assumed 280 participants would receive FSP and 120 would be FSP-eligible but not receive FSP, resulting in 97% power for detecting a mean difference of 5 in HEI-2020 scores, 81% power for detecting a mean difference of 0.25 in acute health care use, and 85% power to detect a standardized effect size of 0.25 for other continuous outcomes. In post hoc power calculations with a sample size of 153 in each group, power was greater than 80% to detect a 16% difference in dichotomized outcomes, 83% for detecting a mean difference of 5 in HEI-2020 scores, 80% for detecting a mean difference of 0.26 in acute health care use, and 80% to detect a standardized effect size of 0.32 for other continuous outcomes.

Two-sided *P* < .05 were considered statistically significant. All data analyses were conducted from August 2024 to April 2025 using SAS, version 9.4 (SAS Institute).

## Results

A total of 153 FSP participant episodes (representing 153 study participants) and 1495 non–FSP participant episodes (610 unique study participants) were included in the sample (Figure). Among 153 FSP participants, 137 (89.5%) enrolled in nutrition and 16 (10.5%) enrolled in housing services (Table 1). The mean (SD) number of months of FSP enrollment was 5.7 (2.9). The most common diagnoses documented on FSP enrollment forms were obesity (88 [57.5%]), depression (42 [27.5%]), and anxiety (28 [18.3%]).

**Table 1.** Characteristics of FSP Participants

Characteristic	Participants, No. (%) (n = 153)
Timing of FSP enrollment	
Between baseline and year 1 assessments	66 (43.1)
Between year 1 and year 2 assessments	41 (26.8)
Between year 2 and year 3 assessments	46 (30.1)
Type of FSP services	
Nutrition: produce vouchers	96 (62.7)
Nutrition: medically tailored meals, food boxes, or education	41 (26.8)
Housing: search and stabilization or legal support	16 (10.5)
No. of months enrolled in FSP, mean (SD)	5.7 (2.9)
Nutrition services	5.8 (2.9)
Housing services	5.1 (3.5)
Documented receipt of intended FSP services	149 (97.4)
Diagnoses documented on FSP enrollment form^a^	
Anxiety, uncontrolled	28 (18.3)
Depression, uncontrolled	42 (27.5)
Diabetes, uncontrolled	19 (12.4)
Hypertension, uncontrolled	16 (10.5)
Obesity (BMI ≥30)	88 (57.5)
Other behavioral health diagnoses^b^	22 (14.4)
Other physical health diagnoses^c^	36 (23.5)
Substance use disorder	23 (15.0)

Abbreviations: BMI, body mass index (calculated as weight in kilograms divided by height in meters squared); FSP, Flexible Services Program.

^a^ More than 1 diagnosis could be documented on FSP enrollment forms.

^b^ Includes bipolar disorder, schizophrenia, and other behavioral health diagnoses.

^c^ Includes asthma, cancer, chronic kidney disease, chronic obstructive pulmonary disease, congestive heart failure, and other physical health diagnoses.

These FSP participants had a mean (SD) age of 43.6 (10.8) years and included 129 females (84.3%) and 24 males (15.7%), of whom 100 (65.4%) self-identified as Hispanic, 11 (7.2%) as non-Hispanic Black, 34 (22.2%) as non-Hispanic White, and 8 (5.2%) as other individuals (Table 2). Prior to FSP participation (time 0), 111 (72.5%) had food insecurity, 68 (44.4%) had housing insecurity, and 55 (35.9%) had both. Of the 610 participants from whom data were obtained for the 1495 non–FSP participant episodes included in the study, 464 (76.1%) were female, 145 (23.8%) were male, and 1 (0.01%) was unknown. Three hundred and eight patients (50.5%) self identified as Hispanic; 57 (9.3%) as non-Hispanic Black; 211 (34.6%) as non-Hispanic White; and 34 (23.4%) as other individuals. The mean (SD) age was 43.2 (11.2) years. After propensity score weighting, the FSP and non-FSP groups had standardized mean differences for all characteristics of 0.08 or lower.

**Table 2.** Characteristics of FSP and Non-FSP Groups

Characteristic	FSP group, No. (%) (n = 153)	Non-FSP group (n = 1495 episodes)^a^		
		Unweighted, No. (%)	Propensity score weighted, No. (%)^b^	Standardized difference
Age, mean (SD), y	43.6 (10.8)	42.5 (11.2)	43.5 (3.4)	0.00
Sex				
Female	129 (84.3)	1152 (77.1)	127 (83.2)	0.03
Male	24 (15.7)	343 (22.9)	26 (16.8)	−0.03
Race and ethnicity^c^				
Hispanic	100 (65.4)	754 (50.4)	101 (65.7)	0.00
Non-Hispanic Black	11 (7.2)	142 (9.5)	12 (7.8)	−0.02
Non-Hispanic White	34 (22.2)	516 (34.5)	34 (21.9)	0.00
Other categories^c^	8 (5.2)	83 (5.6)	7 (4.5)	0.01
Educational level:≤high school	90 (58.8)	678 (45.4)	96 (62.7)	−0.08
Primary language: Spanish	50 (32.7)	289 (19.3)	54 (35.4)	−0.06
Employment status: full or part-time	40 (26.1)	592 (39.6)	44 (28.9)	−0.06
With income <100% FPL	118 (77.1)	962 (64.3)	115 (74.9)	0.05
With ≥1 dependent in household	87 (56.9)	803 (53.7)	85 (55.8)	0.02
With food insecurity	111 (72.5)	718 (48.0)	108 (70.4)	0.04
With housing insecurity	68 (44.4)	458 (30.6)	65 (42.4)	0.04
With both food and housing insecurity	55 (35.9)	307 (20.5)	52 (34.3)	0.04
Used SNAP in past year	117 (76.5)	1083 (72.4)	113 (74.0)	0.06
EHR-documented diagnoses				
Obesity (BMI ≥30)	96 (62.7)	866 (57.9)	96 (63.0)	0.00
Depression	84 (54.9)	698 (46.7)	84 (54.8)	0.00
Anxiety	75 (49.0)	666 (44.5)	74 (48.5)	0.01
Hypertension	56 (36.6)	490 (32.8)	55 (35.8)	0.02
Diabetes	41 (26.8)	276 (18.5)	39 (25.7)	0.03
Other serious mental illness	36 (23.5)	394 (26.4)	35 (22.9)	0.01
Cardiovascular disease	11 (7.2)	91 (6.1)	10 (6.3)	0.03
HEI-2020 score <55^d^	75 (49.0)	789 (52.8)	75 (49.2)	0.00
PSS score ≥15^e^	123 (80.4)	1090 (72.9)	122 (79.8)	0.01
PHQ-8 score ≥10^f^	86 (56.2)	612 (40.9)	82 (53.9)	0.05
GAD-7 score ≥10^g^	82 (53.6)	542 (36.3)	83 (54.5)	0.02
No. of acute hospitalizations and ED visits in past year, mean (SD)	0.12 (0.42)	0.17 (0.89)	0.12 (0.19)	0.02
Health center affiliation				
Center 1	21 (13.7)	223 (14.9)	19 (12.7)	0.03
Center 2	32 (20.9)	298 (19.9)	30 (19.3)	0.04
Center 3	36 (23.5)	401 (26.8)	36 (23.4)	0.00
Center 4	22 (14.4)	315 (21.1)	23 (15.1)	−0.02
Center 5	42 (27.5)	258 (17.3)	45 (29.4)	−0.05

Abbreviations: BMI, body mass index (calculated as weight in kilograms divided by height in meters squared); ED, emergency department; EHR, electronic health record; FPL, federal poverty level; FSP, Flexible Services Program; GAD-7, 7-item Generalized Anxiety Disorder; HEI-2020, Healthy Eating Index; PHQ-8, 8-item Patient Health Questionnaire Depression Scale; PSS, Perceived Stress Scale; SNAP, Supplemental Nutrition Assistance Program.

^a^ Data provided by 610 unique non–FSP participants.

^b^ Inverse probability of treatment weighting was used in propensity score estimates from 1495 surveys (total weight = 153). Propensity score weighting was based on all characteristics in the table along with household size of 3 or more, positive for food insecurity on 2-item screening, elevated financial stress, and self-reported ED visits of more than 2 in the past 12 months.

^c^ Race and ethnicity were self-identified on study surveys. Other categories included self-identified American Indian or Alaska Native, Asian, Native Hawaiian or Pacific Islander, multiracial, and other race (not defined).

^d^ HEI-2020 score range: 0 to 100, with higher scores indicating healthier diet quality.

^e^ PSS score range: 0 to 40, with scores of 14 or higher indicating moderate to severe stress.

^f^ PHQ-8 score range: 0 to 24, with scores of 10 or higher indicating moderate to severe depressive symptoms.

^g^ GAD-7 score range: 0 to 21, with scores of 10 or higher indicating moderate to severe anxiety symptoms.

### Main Outcomes

There was no significant change in the proportion of FSP participants with food insecurity (−1.33%; 95% CI, −9.04% to 6.37%), but there was a decrease in the proportion of non–FSP participants with food insecurity (−6.29%; 95% CI, −8.79% to −3.80%); the difference in changes between groups was 4.96% (95% CI, −3.13% to 13.05%) (Table 3). Results were similar for housing insecurity, with a difference in change of 2.75% (95% CI, −5.39% to 10.88%). Similarly, there were no differences in changes between groups for HEI-2020 scores, PSS scores, and acute hospitalizations and ED visits (Table 3). There were no differences in changes between groups for PHQ-8 and GAD-7 scores (eTable 1 in Supplement 1).

**Table 3.** Changes in Social Needs, Diet, Stress, and Health Care Use Among FSP and Non-FSP Groups^**a**^

Group and change category	FSP enrollment or comparison		Change	Difference in change	*P* value
	Before (time = 0)	After (time = 1 y)			
**With food insecurity, % (95% CI)**					
FSP group (n = 150)	73.33 (66.17 to 80.49)	72.00 (64.73 to 79.27)	−1.33 (−9.04 to 6.37)	4.96 (−3.13 to 13.05)	.37
Non-FSP group (n = 1478)	70.42 (68.09 to 72.75)	64.13 (61.68 to 66.58)	−6.29 (−8.79 to −3.80)		
**With housing insecurity, % (95% CI)**					
FSP group (n = 153)	44.44 (36.48 to 52.41)	40.52 (32.66 to 48.39)	−3.92 (−11.67 to 3.83)	2.75 (−5.39 to 10.88)	.52
Non-FSP group (n = 1492)	42.11 (39.61 to 44.62)	35.45 (33.02 to 37.88)	−6.67 (−9.16 to −4.17)		
**HEI-2020 score, mean (95% CI)^b^**					
FSP group (n = 149)	55.38 (53.14 to 57.62)	55.88 (53.43 to 58.32)	0.50 (−1.91 to 2.91)	0.48 (−2.04 to 3.00)	.72
Non-FSP group (n = 1461)	54.84 (54.12 to 55.57)	54.86 (54.12 to 55.60)	0.02 (−0.73 to 0.76)		
**PSS score, mean (95% CI)^c^**					
FSP group (n = 148)	20.87 (19.64 to 22.09)	20.63 (19.28 to 21.98)	−0.24 (−1.28 to 0.81)	0.34 (−0.75 to 1.43)	.63
Non-FSP group (n = 1459)	20.80 (20.39 to 21.21)	20.22 (19.82 to 20.62)	−0.58 (−0.91 to −0.24)		
**No. of acute hospitalizations and ED visits in past year, mean (95% CI)**					
FSP group (n = 153)	0.12 (0.06 to 0.19)	0.25 (0.13 to 0.36)	0.12 (0.00 to 0.25)	0.11 (−0.02 to 0.24)	.14
Non-FSP group (n = 1495)	0.12 (0.09 to 0.15)	0.13 (0.11 to 0.16)	0.01 (−0.02 to 0.04)		

Abbreviations: FSP, Flexible Services Program; ED, emergency department; HEI-2020, Healthy Eating Index; PSS, Perceived Stress Scale.

^a^ For each outcome, participants with the measure from time 0 and time 1 were analyzed.

^b^ HEI-2020 score range: 0 to 100, with higher scores indicating healthier diet quality.

^c^ PSS score range: 0 to 40, with scores of 14 or higher indicating moderate to severe stress.

### Sensitivity Analyses

In sensitivity analyses restricted to participants enrolled in nutrition services between time 0 and time 1 (n = 137), participants currently receiving nutrition services at time 1 (n = 117, excluding those who had completed nutrition services), and participants who had participated in FSP for 6 months or longer at time 1 (n = 72), the results were similar to those in main analyses (eTables 2, 3, and 4 in Supplement 1). Radar plots of HEI-2020 component scores showed similar patterns for FSP and non-FSP nutrition participants (eFigures 1 and 2 in Supplement 1).

### Qualitative Findings

Interviews were conducted with 27 FSP participants. The themes that emerged from these interviews reflected participants’ mixed experiences; representative quotes are included in Table 4. While some participants expressed relief in food insecurity while receiving nutrition services, others emphasized that the high cost of food was challenging even while receiving services. Participants expressed appreciation for help filling out housing forms but also disappointment with long wait times and limited housing availability. Many nutrition services participants described increased fruit and vegetable intake, but others experienced no dietary changes or returned to less healthy dietary patterns after services ended. Stress often decreased while receiving services, but some participants described increased stress related to difficulties communicating with the SSO’s housing staff. Some participants attributed improvements in their health, such as diabetes and cholesterol control, to FSP, but others suggested that food options should be more tailored to specific health conditions, such as diabetes.

**Table 4.** Qualitative Themes and Representative Quotes From FSP Participants

Themes	Subthemes	Representative quotes
**Food and housing insecurity outcome of FSP**		
Positive change	Relief of food insecurity while receiving services	“Even if for some people, they feel like it’s a little, but for me, it’s, I mean, plenty. Because, sometimes, you have to choose to buy some healthy food or just buy the basics and you cannot get enough fruit or vegetables because everything is going so expensive. So to have this benefit that at least you have a help to provide you with fruit and vegetables, for me, it’s a big, big, big help.” –Produce voucher recipient
	Cost savings and convenience	“Every time I go to the grocery, I know I have that to help me. So it was really convenient.” –Produce voucher recipient
	Appreciation for assistance with filling out housing forms	“They were very good, very efficient. They guided me. They asked me questions like if I needed any help filling out the forms and stuff like that. So they were really good as far as getting the information from me.” –Housing services recipient
Little or no change	High cost of food was a challenge despite services	“It’s not an amount that I say, ‘It’s going to cover me for a whole month and I’m not going to have to look for extra money to eat.’ But I have to put in extra money, because now the basic basket is very, very expensive.” –Produce voucher recipient
	No improvement in housing situation	“What was positive? I thought I was going to get a house. [laughter] I didn’t get it. Still on the waiting list. And [daughter’s name] put all the application, and [SSO staff] says everything’s all set, and I got the letter saying, ‘Nice working with you.’ [laughter] I’m like, ‘Okay.’” –Housing services recipient
**Diet outcome of FSP**		
Positive change	Increased fruit and vegetable intake	“Before we consumed more rice and more junk food, food like that, from outside the home. Now, at least, we rush to make homemade food a little more, because always, if we have vegetables in the house, we only need to prepare a meat, and with that we at least complete the meal. Or we eat more fruit, and it is healthier.” –Produce voucher recipient
	Sustained improvement in diet	“We also kind of got—I got used to the meals. So now, I do [meal delivery program]. So we pay for that, but it was a similar idea where it’s prepackaged. It just helps my life better. That’s what we have all week for dinner. And at work, I make sure to bring my lunch. And I was healthy and lost like over 60 pounds, not just in that time frame alone, but over the course. So it was really helpful.” –Medically tailored meal recipient
Little or no change	No change in diet	“More or less one ate the same, the diet and everything else.” –Food box recipient
	Return to prior diet pattern after services ended	“Yeah. I’ve just been very depressed. I mean, I don’t think I’ve gotten fruit [laughter] or a salad. I just don’t buy anything anymore.” –Produce voucher recipient
**Stress outcome of FSP**		
Positive change	Reduced stress due to nutrition services	“It takes the stress off of me worrying about having enough for this and for that. So yeah, absolutely. I mean, having that and knowing I can get fruits and vegetables? Yeah. That definitely takes off the stress for me.” –Produce voucher recipient
	Reduced stress due to assistance with housing forms	“Yeah. Working with them, yeah. It’s still a stressful situation, but working with them definitely made it feel that there was hope. There’s a light at the end of the dark road, I guess you could say.” –Housing services recipient
Negative change	Increased stress due to housing services	“But it seems like he gave me the brushoff. That’s how I feel. It’s really stressful. I had my hopes up for him putting me in a right direction and doing his job, but he really stressed me out with the job.” –Housing services recipient
	Frustration from difficulties connecting with housing services	“I mean, when I met with her, I did fill out some stuff and signed some stuff. And after that, she said she would be in touch. She was in touch with me. Called me several occasions and we were supposed to meet up again to fill out some referrals or something like that, but it just never happened because it never—it never happened. And at the time, I was homeless. You know what I mean? Sleeping on my truck.” –Housing services recipient
**Health outcome of FSP**		
Positive change	Improved health due to increased fruit and vegetable intake	“In terms of my health, yes, with respect to that, I have had a lot of changes because I use a lot of vegetables and make a lot of green juices. I had high cholesterol and high triglycerides and through those juices, which I am making through this help, they have gone away. I have my health now that I no longer have high cholesterol or high triglycerides, it is normal thanks to God and [produce voucher service].” –Produce voucher recipient
	Improved health due to establishing healthier diet pattern	“I would have never tried certain squashes, but yet I tried it with them. It gave me a better taste of vegetables, and in the long run that worked for me and my diabetes. It made me eat more vegetables than I had before in my life. Like brussels sprouts, I would have never ate them, if I didn’t get them in the box. And so in the long run, my diabetes was much more healthier and much more manageable.” –Food box recipient
Little or no change	No improvement in mental health	“Mental health, I’m the same really. I’m the same. I mean, food’s not really going to take—I mean, well, money-wise, it helps me not worry about—I always have something to eat, you know what I mean? But other than that, my mental health is the same pretty much.” –Produce voucher recipient
	More tailored food options needed for specific health conditions	“It was supposed to help me lower my sugar and stuff. But the stuff they were giving me still was unhealthy, like legumes and other stuff, like beans. It wasn’t really towards the diabetic aspect of my life. It never lowered my sugar. It never helped anything. Because a lot of it was lentils and curries and beans and stuff like that. So it was meant to help me with my diabetes and manage my diabetes, but it didn’t.” –Medically tailored meal recipient

Abbreviations: FSP, Flexible Services Program; SSO, social service organization.

## Discussion

In this prospective cohort of adults from 1 Medicaid ACO, FSP participation was not associated with 1-year changes in quantitative measures of social needs, dietary quality, stress, and acute health care use. Qualitative interviews provided more nuanced perspectives of program effectiveness, with many FSP participants endorsing favorable changes in food insecurity, diet, stress, and health and other participants expressing frustration with, barriers to, and lack of effectiveness of services. The wide range of individual FSP experiences helps to contextualize the null quantitative findings of outcomes averaged across participants.

Most LiveWell/ViveBien study participants had incomes at or below the federal poverty level and were experiencing food or housing insecurity. Given the shortage of affordable housing in Massachusetts,^36^ it is not surprising that FSP was not associated with reduced housing insecurity in the short-term. Interviews revealed that many participants were disappointed and stressed regarding their stagnant housing situation and had difficulty communicating with the housing staff of the SSO. It was, however, unexpected that FSP was not associated with reduced food insecurity. Several prior studies of medically tailored meals or groceries and produce prescriptions have demonstrated reductions in food insecurity,^17,18,20,21,22,37,38,39^ although most had a pre-post design with no comparator.^40^ Interviews with FSP participants revealed factors that may have contributed to the lack of association, especially the high cost of food. Temporary FSP nutrition services were likely not sufficient to resolve food insecurity in the context of steep increases in food prices between 2020 and 2023.^41^ The inability to afford enough food, including healthy food, may also explain the lack of dietary benefits associated with FSP.

Despite evidence that health-related social needs are associated with stress and depressive symptoms,^42,43,44,45,46,47^ few intervention studies measure mental health.^40^ In the LiveWell/ViveBien study, participants had moderately high stress, depression, and anxiety symptoms at baseline, which did not change with FSP. Some FSP participants described how services helped relieve stress, but others revealed their stress was the same or worse. It is likely that participants’ mental health symptoms also had multiple other contributing factors, including preexisting mental health conditions and economic and social factors.

The lack of association between FSP and improved social needs, diet, and stress may partially explain the lack of change in acute health care use. This finding is consistent with some prior studies, including assessments of the Accountable Health Communities Model and the California Medicaid housing program.^13,14,48,49^ An evaluation of 16 health-related social needs pilot programs under California’s Medicaid 1115 waiver found that most were associated with reduced ED visits but not hospitalizations.^48^ Results of the current study differ from findings of an analysis of 17 ACOs that FSP was associated with decreased hospitalizations and ED visits^28^; this discrepancy may be partially explained by differences in how hospitalizations and ED visits (ie, types and periods assessed) and non-FSP comparison groups were defined.

### Limitations

Despite using propensity score weighting and incorporating a wide range of observable social, demographic, and clinical factors, it is possible there was unmeasured confounding, including the potential selection of higher-need ACO beneficiaries into the program. Surveys were not timed precisely around FSP receipt, and 1-year follow-up may have occurred during or just after FSP participation. However, sensitivity analyses of participants currently receiving nutrition services showed no changes associated with FSP. It is also possible that the approximate 6-month exposure was not sufficient for the program benefits to accrue. The study was conducted during the COVID-19 public health emergency, and implementation of other policy changes, including increased Supplemental Nutrition Assistance Program benefits and a housing eviction moratorium,^50,51,52^ may have played a role in the reduced relative benefit of FSP. However, to address time-varying policies and changing COVID-19 pandemic conditions during the study period, propensity weighting balanced the non-FSP and FSP groups on calendar time. It is possible that these policies and other socioeconomic factors may have had larger implications for nonparticipants than participants. The FSP participants in this study were comparable to all MGB ACO FSP enrollees,^27^ but participant characteristics and outcomes may have been different in other ACOs.

## Conclusions

In this cohort study, while a lack of favorable changes in measures of social needs, diet, stress, and health care suggests that FSP might not have met policy objectives, participant interviews demonstrated a range of individual experiences, including improvements in diet, stress, and health as well as challenges in overcoming entrenched social needs. Multisector collaborations, such as FSP, that address only specific needs may have limited effectiveness because they are downstream from the social, political, and economic factors of poor health and health inequities.^53^ Findings of this study underscore the need for ongoing rigorous evaluation of Medicaid and other health system–based social needs interventions to inform future programs.

## Notes

Supplement 1. **eTable 1.** Changes in Depression and Anxiety Scores Among Flexible Services Program and Non-FSP Participants **eTable 2.** Changes in Social Needs, Diet, Stress, and Health Care Use Among Flexible Services Program Nutrition Services Participants (N=137) and Non-FSP Participants **eTable 3.** Changes in Social Needs, Diet, Stress, and Health Care Use Among Current Flexible Services Program Nutrition Services Participants (N=117) and Non-FSP Participants **eTable 4.** Changes in Social Needs, Diet, Stress, and Health Care Use Among Flexible Services Program Participants Receiving FSP Services for 6 Months or More (N=72) and Non-FSP Participants **eFigure 1.** Healthy Eating Index-20 Component Scores for Flexible Services Program Nutrition Services Participants (N=137) and Non-FSP Participants **eFigure 2.** Healthy Eating Index-20 Component Scores for Current Flexible Services Program Nutrition Services Participants (N=117) and Non-FSP Participants

Supplement 2.
Data Sharing Statement

## References

[1] Braveman P, Egerter S, Williams DR. The social determinants of health: coming of age. Annu Rev Public Health. 2011;32:381-398. doi:10.1146/annurev-publhealth-031210-101218 21091195

[2] Dzau VJ, McClellan MB, McGinnis JM, . Vital directions for health and health care: priorities from a National Academy of Medicine Initiative. JAMA. 2017;317(14):1461-1470. doi:10.1001/jama.2017.1964 28324029

[3] National Academies of Sciences, Engineering, and Medicine. Integrating Social Care into the Delivery of Health Care: Moving Upstream to Improve the Nation’s Health. National Academies Press; 2019. 31940159

[4] Horwitz LI, Chang C, Arcilla HN, Knickman JR. Quantifying Health Systems’ investment in social determinants of health, by sector, 2017-19. Health Aff (Millwood). 2020;39(2):192-198. doi:10.1377/hlthaff.2019.01246 32011928

[5] Glied S, D’Aunno T. Health systems and social services-a bridge too far? JAMA Health Forum. 2023;4(8):e233445. doi:10.1001/jamahealthforum.2023.3445 37589971

[6] KFF. Approved and pending section 1115 waivers by state. October 2024. Accessed October 14, 2024. https://www.kff.org/medicaid/issue-brief/medicaid-waiver-tracker-approved-and-pending-section-1115-waivers-by-state/#Table4

[7] Baicker K, McConnell M. Tying innovation to evaluation and accountability in programs to address intersecting health and social needs. JAMA Health Forum. 2022;3(10):e224323. doi:10.1001/jamahealthforum.2022.4323 36218904

[8] Eder M, Henninger M, Durbin S, . Screening and interventions for social risk factors: technical brief to support the US Preventive Services Task Force. JAMA. 2021;326(14):1416-1428. doi:10.1001/jama.2021.12825 34468710 PMC13460784

[9] McCarthy DLC, Horstman C, Bryan A, Shah T. Guide to evidence for health-related social needs interventions: 2022 update. Commonwealth Fund. September 2022. Accessed December 11, 2024. https://www.commonwealthfund.org/sites/default/files/2022-09/ROI_calculator_evidence_review_2022_update_Sept_2022.pdf

[10] Alderwick H, Hutchings A, Briggs A, Mays N. The impacts of collaboration between local health care and non-health care organizations and factors shaping how they work: a systematic review of reviews. BMC Public Health. 2021;21(1):753. doi:10.1186/s12889-021-10630-1 33874927 PMC8054696

[11] Centers for Medicare & Medicaid Services. Accountable health communities model. May 2023. Accessed October 14, 2024. https://www.cms.gov/priorities/innovation/innovation-models/ahcm

[12] Renaud J, McClellan SR, DePriest K, . Addressing health-related social needs via community resources: lessons from accountable health communities. Health Aff (Millwood). 2023;42(6):832-840. doi:10.1377/hlthaff.2022.01507 37196207

[13] Parish W, Beil H, He F, . Health care impacts of resource navigation for health-related social needs in the accountable health communities model. Health Aff (Millwood). 2023;42(6):822-831. doi:10.1377/hlthaff.2022.01502 37196210

[14] Knox MJ, Hernandez EA, Ahern J, . Rental housing deposits and health care use. JAMA Health Forum. 2024;5(9):e242802. doi:10.1001/jamahealthforum.2024.2802 39240580 PMC11380099

[15] Leung CW, Odoms-Young A, Essel K. Food insecurity is a source of toxic stress. JAMA Pediatr. 2024;178(4):327-328. doi:10.1001/jamapediatrics.2023.6400 38315497

[16] Volpp KG, Berkowitz SA, Sharma SV, ; American Heart Association. Food is medicine: a presidential advisory from the American Heart Association. Circulation. 2023;148(18):1417-1439. doi:10.1161/CIR.0000000000001182 37767686 PMC12168720

[17] Berkowitz SA, Delahanty LM, Terranova J, . Medically tailored meal delivery for diabetes patients with food insecurity: a randomized cross-over trial. J Gen Intern Med. 2019;34(3):396-404. doi:10.1007/s11606-018-4716-z 30421335 PMC6420590

[18] Seligman HK, Smith M, Rosenmoss S, Marshall MB, Waxman E. Comprehensive diabetes self-management support from food banks: a randomized controlled trial. Am J Public Health. 2018;108(9):1227-1234. doi:10.2105/AJPH.2018.304528 30024798 PMC6085038

[19] Ferrer RL, Neira LM, De Leon Garcia GL, Cuellar K, Rodriguez J. Primary care and food bank collaboration to address food insecurity: a pilot randomized trial. Nutr Metab Insights. 2019;12:1178638819866434. doi:10.1177/1178638819866434 31384130 PMC6664622

[20] Hager K, Du M, Li Z, . Impact of produce prescriptions on diet, food security, and cardiometabolic health outcomes: a multisite evaluation of 9 produce prescription programs in the United States. Circ Cardiovasc Qual Outcomes. 2023;16(9):e009520. doi:10.1161/CIRCOUTCOMES.122.009520 37641928 PMC10529680

[21] Ridberg RA, Bell JF, Merritt KE, Harris DM, Young HM, Tancredi DJ. Effect of a fruit and vegetable prescription program on children’s fruit and vegetable consumption. Prev Chronic Dis. 2019;16:E73. doi:10.5888/pcd16.180555 31198165 PMC6583818

[22] Ridberg RA, Marpadga S, Akers MM, Bell JF, Seligman HK. Fruit and vegetable vouchers in pregnancy: preliminary impact on diet & food security. J Hunger Environ Nutr. 2021;16(2):149-163. doi:10.1080/19320248.2020.1778593

[23] Trapl ES, Smith S, Joshi K, . Dietary impact of produce prescriptions for patients with hypertension. Prev Chronic Dis. 2018;15:E138. doi:10.5888/pcd15.180301 30447106 PMC6266424

[24] Ryan AM, Kutob RM, Suther E, Hansen M, Sandel M. Pilot study of impact of medical-legal partnership services on patients’ perceived stress and wellbeing. J Health Care Poor Underserved. 2012;23(4):1536-1546. doi:10.1353/hpu.2012.0179 23698668

[25] Rosen Valverde JN, Backstrand J, Hills L, Tanuos H. Medical-legal partnership impact on parents’ perceived stress: a pilot study. Behav Med. 2019;45(1):70-77. doi:10.1080/08964289.2018.1481011 29944063

[26] Lindau ST, Makelarski JA, Abramsohn EM, . CommunityRx: a real-world controlled clinical trial of a scalable, low-intensity community resource referral intervention. Am J Public Health. 2019;109(4):600-606. doi:10.2105/AJPH.2018.304905 30789775 PMC6417580

[27] McCurley JL, Fung V, Levy DE, . Assessment of the Massachusetts Flexible Services Program to address food and housing insecurity in a Medicaid accountable care organization. JAMA Health Forum. 2023;4(6):e231191. doi:10.1001/jamahealthforum.2023.1191 37266960 PMC10238945

[28] Hager K, Sabatino M, Williams J, . Medicaid nutrition supports associated with reductions in hospitalizations and ED visits in Massachusetts, 2020-23. Health Aff (Millwood). 2025;44(4):413-421. doi:10.1377/hlthaff.2024.01409 40193848

[29] Automated Self-Administered 24-Hour (ASA24) dietary assessment tool. 2024. Accessed November 15, 2024. https://epi.grants.cancer.gov/asa24/

[30] Cohen S, Williamson GM. Perceived stress in a probability sample of the United States. In: Spacaman S, Oskamp S, eds. The Social Psychology of Health. Sage; 1988:31-67.

[31] Kroenke K, Strine TW, Spitzer RL, Williams JB, Berry JT, Mokdad AH. The PHQ-8 as a measure of current depression in the general population. J Affect Disord. 2009;114(1-3):163-173. doi:10.1016/j.jad.2008.06.026 18752852

[32] Spitzer RL, Kroenke K, Williams JB, Löwe B. A brief measure for assessing generalized anxiety disorder: the GAD-7. Arch Intern Med. 2006;166(10):1092-1097. doi:10.1001/archinte.166.10.1092 16717171

[33] Shams-White MM, Pannucci TE, Lerman JL, . Healthy Eating Index-2020: review and update process to reflect the dietary guidelines for Americans, 2020-2025. J Acad Nutr Diet. 2023;123(9):1280-1288. doi:10.1016/j.jand.2023.05.015 37201748 PMC10524328

[34] Gale NK, Heath G, Cameron E, Rashid S, Redwood S. Using the framework method for the analysis of qualitative data in multi-disciplinary health research. BMC Med Res Methodol. 2013;13:117. doi:10.1186/1471-2288-13-117 24047204 PMC3848812

[35] Austin PC. An introduction to propensity score methods for reducing the effects of confounding in observational studies. Multivariate Behav Res. 2011;46(3):399-424. doi:10.1080/00273171.2011.568786 21818162 PMC3144483

[36] National Low Income Housing Coalition. Housing needs by state/Massachusetts. Accessed October 27, 2024. https://nlihc.org/housing-needs-by-state/massachusetts

[37] Palar K, Napoles T, Hufstedler LL, . Comprehensive and medically appropriate food support is associated with improved HIV and diabetes health. J Urban Health. 2017;94(1):87-99. doi:10.1007/s11524-016-0129-7 28097614 PMC5359179

[38] Cheyne K, Smith M, Felter EM, . Food bank-based diabetes prevention intervention to address food security, dietary intake, and physical activity in a food-insecure cohort at high risk for diabetes. Prev Chronic Dis. 2020;17:E04. doi:10.5888/pcd17.190210 31922370 PMC6977780

[39] Aiyer JN, Raber M, Bello RS, . A pilot food prescription program promotes produce intake and decreases food insecurity. Transl Behav Med. 2019;9(5):922-930. doi:10.1093/tbm/ibz112 31570927 PMC6768858

[40] Downer S, Clippinger E, Kummer C, Hager K, Acosta V. Food is Medicine research action plan. Aspen Institute; 2022. Accessed October 18, 2024. https://www.aspeninstitute.org/wp-content/uploads/2022/01/Food-is-Medicine-Action-Plan-Final_012722.pdf

[41] US Department of Agriculture Economic Research Service. Summary findings: food price outlook, 2024 and 2025. Accessed October 27, 2024. https://www.ers.usda.gov/data-products/food-price-outlook/summary-findings/

[42] Arenas DJ, Thomas A, Wang J, DeLisser HM. A systematic review and meta-analysis of depression, anxiety, and sleep disorders in US adults with food insecurity. J Gen Intern Med. 2019;34(12):2874-2882. doi:10.1007/s11606-019-05202-4 31385212 PMC6854208

[43] Pourmotabbed A, Moradi S, Babaei A, . Food insecurity and mental health: a systematic review and meta-analysis. Public Health Nutr. 2020;23(10):1778-1790. doi:10.1017/S136898001900435X 32174292 PMC10200655

[44] Nagata JM, Ganson KT, Whittle HJ, . Food insufficiency and mental health in the US during the COVID-19 pandemic. Am J Prev Med. 2021;60(4):453-461. doi:10.1016/j.amepre.2020.12.004 33602534 PMC9067067

[45] Taylor MP, Pevalin DJ, Todd J. The psychological costs of unsustainable housing commitments. Psychol Med. 2007;37(7):1027-1036. doi:10.1017/S0033291706009767 17224094

[46] Singh A, Daniel L, Baker E, Bentley R. Housing disadvantage and poor mental health: a systematic review. Am J Prev Med. 2019;57(2):262-272. doi:10.1016/j.amepre.2019.03.018 31326010

[47] Dobbins SK, Garcia CM, Evans JL, Valle K, Guzman D, Kushel MB. Continued homelessness and depressive symptoms in older adults. JAMA Netw Open. 2024;7(8):e2427956. doi:10.1001/jamanetworkopen.2024.27956 39145977 PMC11327886

[48] Chuang E, Yue D, O’Masta B, Haley LA, Zhou W, Pourat N. Program implementation strategies associated with reduced acute care utilization for Medicaid beneficiaries in California’s Whole Person Care pilot program. Med Care Res Rev. 2024;81(6):432-443. doi:10.1177/10775587241273404 39225361 PMC11528969

[49] Arbour M, Fico P, Atwood S, . Primary care-based housing program reduced outpatient visits; patients reported mental and physical health benefits. Health Aff (Millwood). 2024;43(2):200-208. doi:10.1377/hlthaff.2023.01046 38315923

[50] Baicker K. How much safety did the social safety net provide during the COVID-19 pandemic? JAMA Health Forum. 2023;4(10):e234237. doi:10.1001/jamahealthforum.2023.4237 37824156

[51] Ali AK, Wehby GL. State eviction moratoriums during the COVID-19 pandemic were associated with improved mental health among people who rent. Health Aff (Millwood). 2022;41(11):1583-1589. doi:10.1377/hlthaff.2022.00750 36343323

[52] Richterman A, Roberto CA, Thirumurthy H. Associations between ending Supplemental Nutrition Assistance Program emergency allotments and food insufficiency. JAMA Health Forum. 2023;4(8):e232511. doi:10.1001/jamahealthforum.2023.2511 37566430 PMC10422192

[53] Berkowitz SA. Multisector collaboration vs. social democracy for addressing social determinants of health. Milbank Q. 2024;102(2):280-301. doi:10.1111/1468-0009.12685 38156764 PMC11176409

