# Supplementary material for: Food and Housing Insecurity, Stress, and Health Care Use After Medicaid Expanded Services Program
Source: JAMA Netw Open. 2025 Jul 8;8(7):e2519507. doi: 10.1001/jamanetworkopen.2025.19507 (PMC12238900; doi:10.1001/jamanetworkopen.2025.19507)
Supplement: Supplement 1. — eTable 1. Changes in Depression and Anxiety Scores Among Flexible Services Program and Non-FSP Participants eTable 2. Changes in Social Needs, Diet, Stress, and Health Care Use Among Flexible Services Program Nutrition Services Participants (N=137) and Non-FSP Participants eTable 3. Changes in Social Needs, Diet, Stress, and Health Care Use Among Current Flexible Services Program Nutrition Services Participants (N=117) and Non-FSP Participants eTable 4. Changes in Social Needs, Diet, Stress, and Health Care Use Among Flexible Services Program Participants Receiving FSP Services for 6 Months or More (N=72) and Non-FSP Participants eFigure 1. Healthy Eating Index-20 Component Scores for Flexible Services Program Nutrition Services Participants (N=137) and Non-FSP Participants eFigure 2. Healthy Eating Index-20 Component Scores for Current Flexible Services Program Nutrition Services Participants (N=117) and Non-FSP Participants [file jamanetwopen-e2519507-s001.pdf]

Supplemental Online Content

Thorndike AN, McCurley JL, Chang Y, et al. Food and housing insecurity, stress, and health care use after Medicaid expanded services program. *JAMA Netw Open*. 2025;8(7):e2519507. doi:10.1001/jamanetworkopen.2025.19507

eTable 1. Changes in Depression and Anxiety Scores Among Flexible Services Program and Non-FSP Participants

eTable 2. Changes in Social Needs, Diet, Stress, and Health Care Use Among Flexible Services Program Nutrition Services Participants (N=137) and Non-FSP Participants

eTable 3. Changes in Social Needs, Diet, Stress, and Health Care Use Among Current Flexible Services Program Nutrition Services Participants (N=117) and Non-FSP Participants

eTable 4. Changes in Social Needs, Diet, Stress, and Health Care Use Among Flexible Services Program Participants Receiving FSP Services for 6 Months or More (N=72) and Non-FSP Participants

eFigure 1. Healthy Eating Index-20 Component Scores for Flexible Services Program Nutrition Services Participants (N=137) and Non-FSP Participants

eFigure 2. Healthy Eating Index-20 Component Scores for Current Flexible Services Program Nutrition Services Participants (N=117) and Non-FSP Participants

This supplemental material has been provided by the authors to give readers additional information about their work.

© 2025 Thorndike AN et al. *JAMA Network Open*

eTable 1. Changes in depression and anxiety scores among Flexible Services Program and non-FSP participants.

Group and change category	FSP enrollment (or comparison)		Change	Difference in change	*P* value
	Before (time = 0)	After (time = 1 y)			
PHQ-8 score,[a] mean (95% CI)					
FSP group (n=150)	11.08 (10.01, 12.15)	10.83 (9.74, 11.91)	-0.25 (-1.12, 0.62)	-0.05 (-0.97, 0.87)	0.92
Non-FSP group (n=1469)	10.55 (10.22, 10.89)	10.35 (10.01, 10.68)	-0.20 (-0.51, 0.10)		
GAD-7 score,[b] mean (95% CI)					
FSP group (n=150)	10.11 (9.10, 11.12)	9.45 (8.42, 10.48)	-0.67 (-1.66, 0.32)	0.24 (-0.79, 1.27)	0.72
Non-FSP group (n=1474)	9.93 (9.61, 10.24)	9.02 (8.69, 9.34)	-0.91 (-1.20, -0.62)		

FSP: Flexible Services Program; PHQ-8: Patient Health Questionnaire-8; GAD-7: Generalized Anxiety Disorder-7
For each outcome, participants with measures from Time 0 and Time 1 were analyzed.
[a] PHQ-8 score, range, 0-24, scores ≥10 indicated moderate to severe depressive symptoms.
[b] GAD-7 score, range 0-21, scores ≥10 indicated moderate to severe anxiety symptoms.

© 2025 Thorndike AN et al. *JAMA Network Open*

eTable 2. Changes in social needs, diet, stress, and health care use among Flexible Services Program nutrition services participants (N=137) and non-FSP participants.

Group and change category	FSP enrollment (or comparison)		Change	Difference in change	*P* value
	Before (time = 0)	After (time = 1 y)			
With food insecurity, % (95% CI)					
FSP group (n=134)	73.13 (65.53, 80.74)	72.39 (64.72, 80.06)	-0.75 (-8.72, 7.23)	2.91 (-5.47, 11.30)	0.65
Non-FSP group (n=1478)	69.33 (66.97, 71.68)	65.66 (63.24, 68.09)	-3.66 (-6.25, -1.07)		
With housing insecurity, % (95% CI)					
FSP group (n=137)	42.34 (33.96, 50.71)	38.69 (30.43, 46.94)	-3.65 (-11.95, 4.65)	2.20 (-6.47, 10.87)	0.63
Non-FSP group (n=1492)	39.40 (36.92, 41.89)	33.55 (31.15, 35.95)	-5.85 (-8.39, -3.31)		
HEI-20 score,[a] mean (95% CI)					
FSP group (n=134)	56.03 (53.69, 58.37)	56.54 (54.01, 59.06)	0.51 (-2.02, 3.04)	0.45 (-2.18, 3.08)	0.76
Non-FSP group (n=1461)	55.25 (54.53, 55.97)	55.31 (54.56, 56.05)	0.06 (-0.69, 0.81)		
PSS score,[b] mean (95% CI)					
FSP group (n=134)	20.86 (19.57, 22.15)	20.60 (19.21, 22.00)	-0.25 (-1.34, 0.83)	0.13 (-1.00, 1.27)	0.87
Non-FSP group (n=1459)	20.68 (20.27, 21.09)	20.29 (19.89, 20.70)	-0.39 (-0.72, -0.06)		
No. of acute hospitalizations and ED visits in past year, mean (95% CI)					
FSP group (n=137)	0.09 (0.04, 0.15)	0.20 (0.11, 0.30)	0.11 (0.00, 0.22)	0.08 (-0.04, 0.19)	0.36
Non-FSP group (n=1495)	0.08 (0.06, 0.10)	0.11 (0.09, 0.13)	0.06 (0.03, 0.09)		

FSP: Flexible Services Program; ED: emergency department; HEI-20: Healthy Eating Index 2020; PSS: Perceived Stress Scale.
For each outcome, participants with measures from Time 0 and Time 1 were analyzed.
[a] HEI-20 score, range 0-100, higher score indicates healthier diet quality.
[b] PSS score, range 0-40, scores ≥14 indicate moderate to severe stress.

© 2025 Thorndike AN et al. *JAMA Network Open*

eTable 3. Changes in social needs, diet, stress, and health care use among current Flexible Services Program nutrition services participants (N=117) and non-FSP participants.

Group and change category	FSP enrollment (or comparison)		Change	Difference in change	*P* value
	Before (time = 0)	After (time = 1 y)			
With food insecurity, % (95% CI)					
FSP group (n=114)	70.18 (61.65, 78.70)	71.05 (62.60, 79.51)	0.88 (-8.19, 9.95)	5.98 (-3.43, 15.38)	0.37
Non-FSP group (n=1478)	66.20 (63.78, 68.61)	61.10 (58.61, 63.59)	-5.10 (-7.62, -2.58)		
With housing insecurity, % (95% CI)					
FSP group (n=117)	42.74 (33.64, 51.83)	37.61 (28.70, 46.51)	-5.13 (-13.05, 2.79)	2.32 (0.00, 10.59)	0.59
Non-FSP group (n=1492)	40.10 (37.61, 42.59)	32.65 (30.27, 35.03)	-7.45 (-9.87, -5.02)		
HEI-20 score,[a] mean (95% CI)					
FSP group (n=115)	55.70 (53.11, 58.28)	57.12 (54.35, 59.89)	1.42 (-1.44, 4.28)	1.40 (-1.55, 4.35)	0.43
Non-FSP group (n=1461)	55.95 (55.18, 56.71)	55.96 (55.24, 56.69)	0.02 (-0.71, 0.74)		
PSS score,[b] mean (95% CI)					
FSP group (n=113)	20.63 (19.22, 22.04)	20.14 (18.61, 21.67)	-0.49 (-1.70, 0.72)	0.40 (-0.85, 1.66)	0.64
Non-FSP group (n=1459)	20.26 (19.84, 20.69)	19.37 (18.97, 19.78)	-0.89 (-1.24, -0.54)		
No. of acute hospitalizations and ED visits in past year, mean (95% CI)					
FSP group (n=117)	0.12 (0.04, 0.20)	0.27 (0.13, 0.40)	0.15 (-0.01, 0.31)	0.12 (-0.04, 0.28)	0.27
Non-FSP group (n=1495)	0.10 (0.07, 0.13)	0.13 (0.10, 0.15)	0.06 (0.03, 0.09)		

FSP: Flexible Services Program; ED: emergency department; HEI-20: Healthy Eating Index 2020; PSS: Perceived Stress Scale.
For each outcome, participants with measures from Time 0 and Time 1 were analyzed.
[a] HEI-20 score, range 0-100, higher score indicates healthier diet quality.
[b] PSS score, range 0-40, scores ≥14 indicate moderate to severe stress.

© 2025 Thorndike AN et al. *JAMA Network Open*

eTable 4. Changes in social needs, diet, stress, and health care use among Flexible Services Program participants receiving FSP services for 6 months or more (N=72) and non-FSP participants.

Group and change category	FSP enrollment (or comparison)		Change	Difference in change	*P* value
	Before (time = 0)	After (time = 1 y)			
With food insecurity, % (95% CI)					
FSP group (n=71)	80.28 (70.80, 89.77)	80.28 (70.80, 89.77)	0.00 (-12.00, 12.00)	9.22 (-3.01, 21.45)	0.29
Non-FSP group (n=1478)	76.50 (74.34, 78.67)	67.28 (64.88, 69.67)	-9.22 (-11.62, -6.82)		
With housing insecurity, % (95% CI)					
FSP group (n=72)	43.06 (31.34, 54.77)	44.44 (32.69, 56.20)	1.39 (-10.10, 12.88)	8.20 (-3.56, 19.96)	0.23
Non-FSP group (n=1492)	42.04 (39.53, 44.54)	35.22 (32.80, 37.65)	-6.81 (-9.35, -4.28)		
HEI-20 score,[a] mean (95% CI)					
FSP group (n=70)	57.29 (54.17, 60.40)	55.97 (52.37, 59.56)	-1.32 (-4.67, 2.03)	-0.64 (-4.06, 2.78)	0.77
Non-FSP group (n=1461)	55.68 (55.00, 56.36)	54.99 (54.28, 55.71)	-0.68 (-1.40, 0.03)		
PSS score,[b] mean (95% CI)					
FSP group (n=71)	22.14 (20.63, 23.65)	21.32 (19.77, 22.88)	-0.82 (-2.12, 0.48)	0.06 (-1.28, 1.40)	0.96
Non-FSP group (n=1459)	22.22 (21.82, 22.63)	21.35 (20.94, 21.76)	-0.88 (-1.19, -0.56)		
No. of acute hospitalizations and ED visits in past year, mean (95% CI)					
FSP group (n=72)	0.11 (0.03, 0.20)	0.19 (0.03, 0.36)	0.08 (-0.09, 0.25)	0.07 (-0.11, 0.24)	0.60
Non-FSP group (n=1495)	0.10 (0.08, 0.13)	0.12 (0.10, 0.15)	0.06 (0.03, 0.09)		

FSP: Flexible Services Program; ED: emergency department; HEI-20: Healthy Eating Index 2020; PSS: Perceived Stress Scale.
For each outcome, participants with measures from Time 0 and Time 1 were analyzed.
[a] HEI-20 score, range 0-100, higher score indicates healthier diet quality.
[b] PSS score, range 0-40, scores ≥14 indicate moderate to severe stress.

© 2025 Thorndike AN et al. *JAMA Network Open*

eFigure 1. Healthy Eating Index-20 component scores for Flexible Services Program nutrition services participants (N=137) and non-FSP participants.

A. FSP nutrition services group

B. Non-FSP group

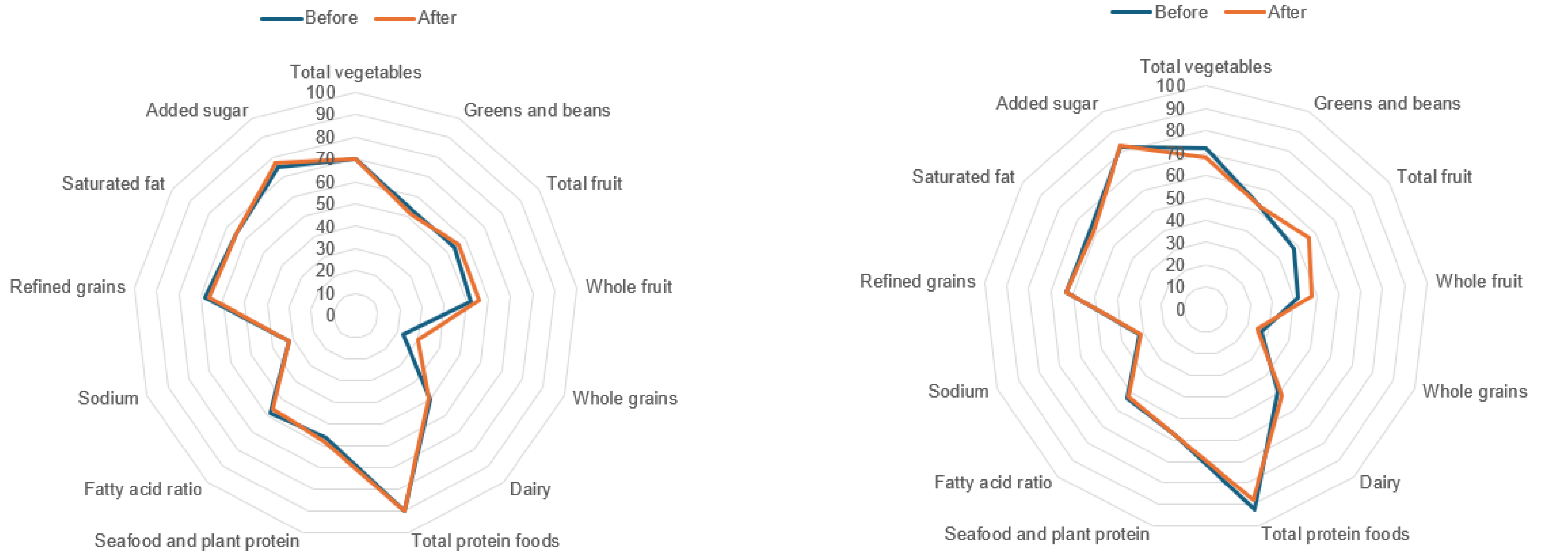

Before: T0, before receiving Flex nutrition services.
After: T1, during or after receipt of Flex nutrition service.

© 2025 Thorndike AN et al. *JAMA Network Open*

eFigure 2. Healthy Eating Index-20 component scores for current Flexible Services Program nutrition services participants (N=117) and non-FSP participants.

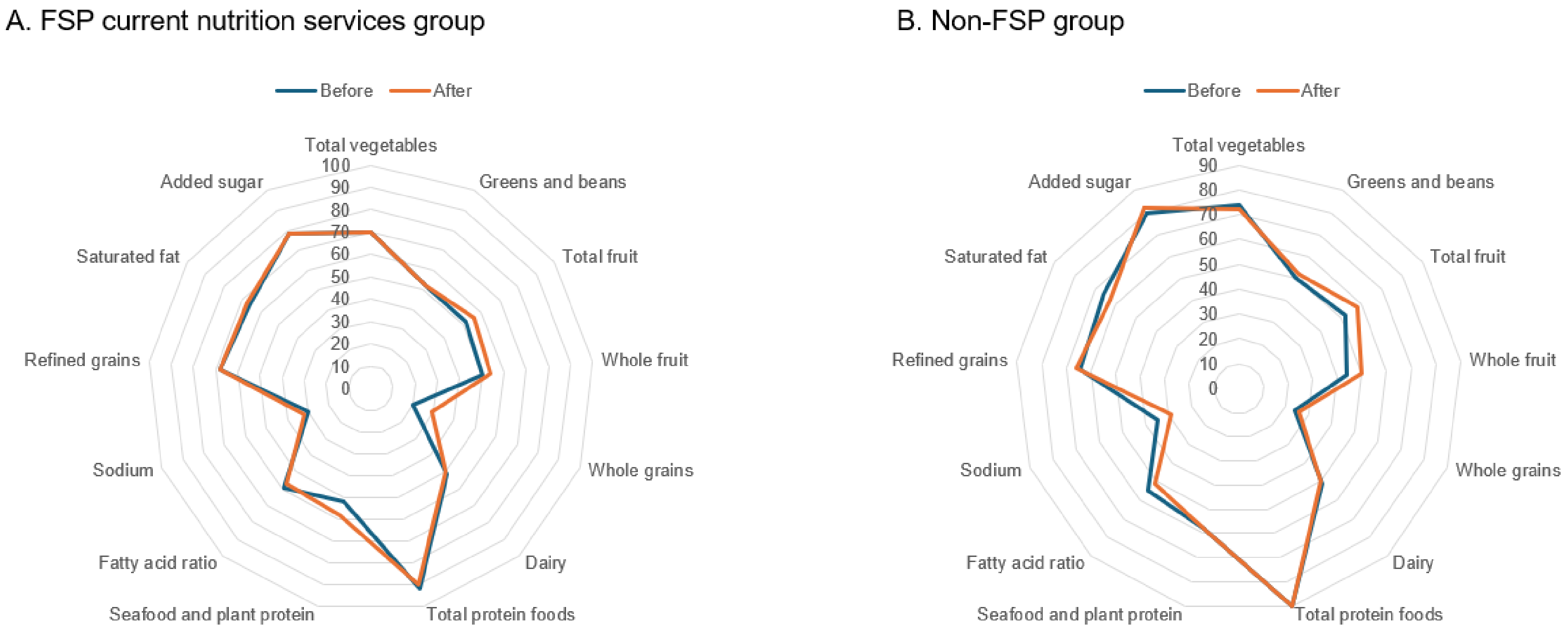

Before: T0, before receiving Flex nutrition services.
During: T1, during receipt of Flex nutrition service.

© 2025 Thorndike AN et al. *JAMA Network Open*
