# Supplementary material for: Food and Housing Insecurity, Stress, and Health Care Use After Medicaid Expanded Services Program
Source: JAMA Netw Open. 2025 Jul 8;8(7):e2519507. doi: 10.1001/jamanetworkopen.2025.19507 (PMC12238900; doi:10.1001/jamanetworkopen.2025.19507)
Supplement: Supplement 2. — Data Sharing Statement [file jamanetwopen-e2519507-s002.pdf]

Data Sharing Statement

Thorndike. Food and Housing Insecurity, Stress, and Health Care Use After Medicaid Expanded Services Program. *JAMA Netw Open*. Published July 08, 2025. doi:10.1001/jamanetworkopen.2025.19507

Data
Data available: Yes
Data types: Deidentified participant data
How to access data: Data will be available upon request by emailing athorndike@mgh.harvard.edu.
When available: With publication

Supporting Documents
Document types: Data dictionary
How to access documents: Data dictionary will be made available together with the data on request by emailing athorndike@mgh.harvard.edu.
When available: With publication

Additional Information
Who can access the data: Data will be available to researchers whose proposed use of the data has been approved.
Types of analyses: Data will be available for any research-related purpose after review by the study team.
Mechanisms of data availability: Data will be made available after a signed data use agreement.
Any additional restrictions: MassHealth may have additional restrictions on which data collected prior to April 2023 can be made available to other researchers.
